# Supplementary figures and images for: lncRNA TMEM51-AS1 and RUSC1-AS1 function as ceRNAs for induction of laryngeal squamous cell carcinoma and prediction of prognosis
Source: PeerJ. 2019 Sep 10;7:e7456. doi: 10.7717/peerj.7456 (PMC6743450; doi:10.7717/peerj.7456)

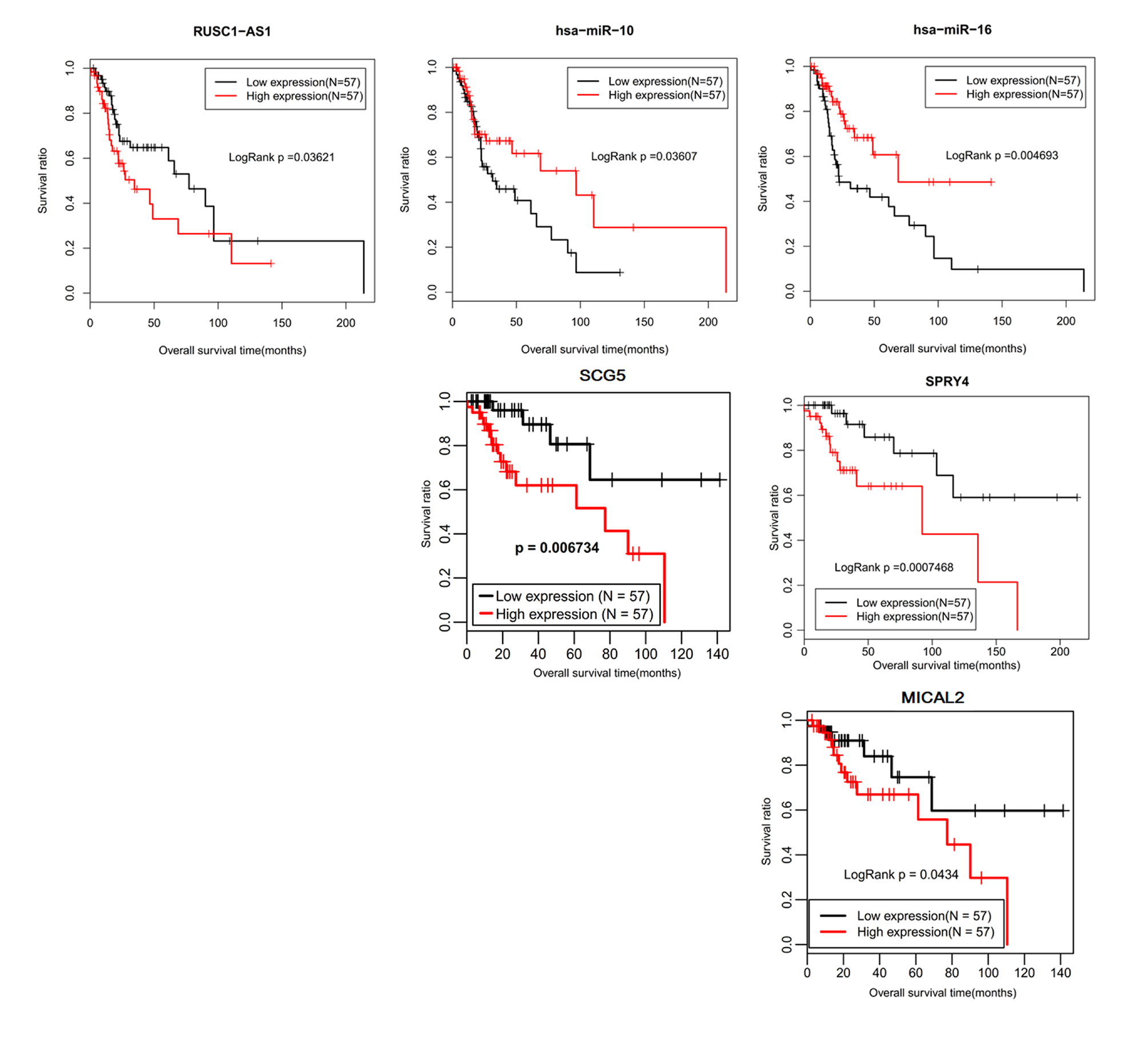

Supplement: Figure S1 [file peerj-07-7456-s010.png]

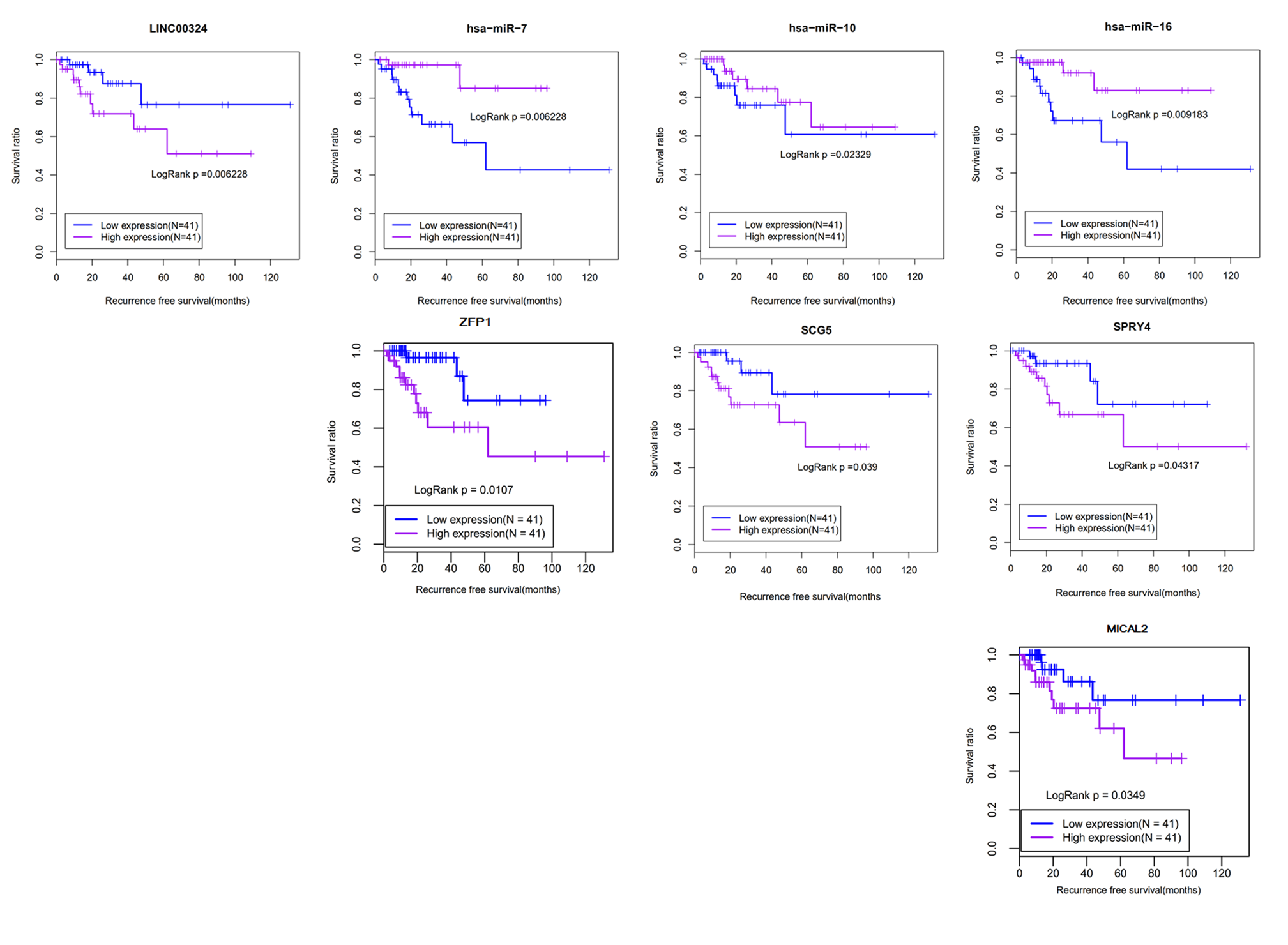

Supplement: Figure S2 [file peerj-07-7456-s011.png]
